# Predictive Value of Positive Margins in Diagnostic Biopsies of Dysplastic Nevi

**DOI:** 10.1155/2020/6716145

**Published:** 2020-01-29

**Authors:** Jeffrey S. Dickman, Reem M. Haddad, Andrew Racette

**Affiliations:** ^1^HonorHealth Internal Medicine Residency, Scottsdale, AZ 85255, USA; ^2^KCU-GMEC Phoenix Dermatology Program, Mesa, AZ 85206, USA

## Abstract

Dysplastic nevi (DN) are common and controversial and the best choice for management of DN after diagnosis is not always clear. The presence of positive margins found on diagnostic biopsy is used by many dermatologists when deciding whether to re-excise these lesions. In order to quantify the predictive value of positive margins in diagnostic biopsies of DN, we performed a review and analysis of the concordance between the histological findings of biopsies and their subsequent excisions. A total of 122 pathology reports from diagnostic biopsies of DN with nevus cells present at the tissue margin were reviewed. Within this sample, 68 total postbiopsy excisions had been performed. The excisional pathology reports were reviewed for the presence of residual or recurrent nevus cells. Residual nevus cells were reported in 29 of 63 available excisional pathology reports illustrating a positive predictive value (PPV) of positive margins in diagnostic biopsies of DN of 46.0%. We present this value along with PPVs from the very few existing similar studies. The quantified predictive value of positive margins in diagnostic biopsies is useful information for providers who must make decisions regarding the best treatment options for patients with DN. The low PPV of positive margins lends further evidence that DN of moderate severity or less may simply be monitored.

## 1. Introduction

Dysplastic nevi (DN) are common, but their behavior and biology is controversial. The best choice for management of DN after diagnosis is not always clear. The presence of positive margins found on diagnostic biopsy, as indicated by pathology reports, is used by many dermatologists when deciding whether to re-excise these lesions [1]. However, it is not uncommon that after a subsequent excision is performed, no residual nevus cells are detected within the excised tissue. This is unexpected given the initially positive diagnostic margin. The degree of concordance of diagnostic biopsies with positive tissue margins and their subsequent excisions can be quantified and reported as the positive predictive value (PPV).

Few studies have evaluated this metric until now. In some, the PPV is calculated directly by the study authors. In others, we have calculated the PPV using the authors' presented data. Results of the five known studies in which the PPV of positive margins in diagnostic biopsies of DN has been calculated or may be calculated using the study's data are included as follows. Until now, no single article or review has included all of this data nor standardized it through the use of the PPV.

In a retrospective study, Maghari found that of 236 shave biopsies showing DN with moderate or severe atypia and positive margins, 136 were found to be positive for residual or recurrent nevus cells on excision [2]. This demonstrates a PPV of 44.9%.

A study by Reddy et al. found that upon re-excision of 127 margin-positive biopsies, 42 showed nevus cells, demonstrating a PPV of 33% [3].

Strazzula et al. found that following 405 margin-positive biopsies, 90 re-excisions contained nevus cells, a PPV of 18.2%. They expounded on their findings stating “the histopathological yield from re-excising margin-positive mildly and moderately DN is low, and the benefit of ensuring complete nevus clearance may not offset the potential risk to the patient and cost associated with an additional procedure [4].”

According to data presented by Cohen et al., when a single lateral margin of the diagnostic biopsy was positive, a PPV of 24.0% was demonstrated. When both lateral diagnostic margins were positive, the PPV increased to 39.7% [5].

Most recently, Duncan et al. reported a PPV of 29.41% in DN with moderate atypia or greater [6].

In our study we quantify the PPV of positive margins in diagnostic biopsies of DN by performing a retrospective review and analysis of the concordance between the histological findings of biopsies and subsequent excisions in our own patient population. We present this PPV in comparison and addition to the five above studies.

Quantifying the PPV of positive margins in diagnostic biopsies of DN, and presenting this information with findings from other studies, will give providers more information when making treatment decisions for their patients with biopsy-proven, margin-positive DN.

## 2. Materials and Methods

Using an institutional review board-approved method, the diagnostic biopsy histopathology records from a community-based dermatology practice were reviewed for reports of DN.

The methods for diagnosis and treatment of DN in this practice typically included a diagnostic shave biopsy with intent to remove the entire visible lesion, followed by possible excision. The decision for postbiopsy excision was determined by both the degree of melanocytic atypia and the presence of nevus cells at the margin of the diagnostic specimen as indicated by the pathology report. DN with reported mild atypia were typically not excised and were monitored clinically for recurrence. Excision in this group was reserved for lesions that appeared especially clinically concerning, disproportionate to their microscopic diagnosis. DN with moderate atypia were typically excised if positive margins were found in the initial biopsy and were otherwise monitored. DN with severe atypia were excised regardless of biopsy margin status.

The processing and sectioning of biopsy specimens was performed by an outside private dermatopathology group using the usual bread-loafing manner. Each of the dermatopathologists reading slides for this practice routinely commented on the margin status of pigmented lesion biopsy specimens.

A total of 122 diagnostic biopsies of DN with nevus cells present at the tissue margin collected between January 1 and December 31, 2017, were available for review. Within this sample, 68 total postbiopsy excisions had been performed. The histopathological findings on initial biopsy specified degrees of cytologic atypia including slight or mild, mild-moderate, moderate, moderate-severe, severe, and other. In the “other” category, no specific degree of atypia was mentioned, but instead terms such as “bizarre cytologic atypia,” “with atypical features,” or simply “atypical” were used.

Excisional concordance with initial biopsy was determined through review of excisional histopathology reports. If the presence of nevus cells was reported in the excised tissue, the excision was deemed concordant with its margin-positive biopsy and thus considered a true positive (TP). If no nevus cells were reported, the biopsy and excision were deemed discordant and therefore false positive (FP). After quantifying TP and FP results, PPV was calculated as follows:

(1) $$\begin{array}{c} \text{PPV}=\frac{\text{TP}}{\left(\text{TP}+\text{FP}\right)}. \end{array}$$

Two excisional reports were also found to have discordant diagnoses compared to their respective diagnostic biopsies. One biopsy which was originally reported as a moderately DN was reported as moderate-severe on excision. Another biopsy originally reported as a moderately DN was reported as malignant melanoma in situ following excision.

## 3. Results and Discussion

**Figure 1:**
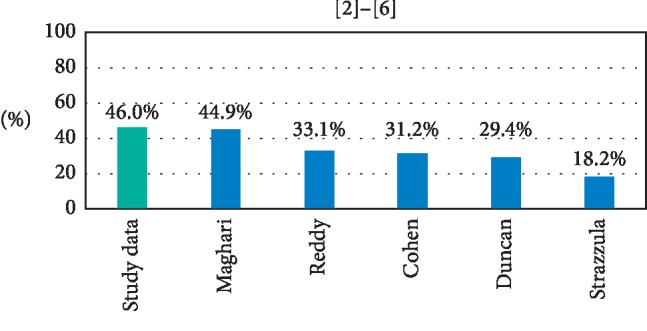
PPV of positive margins in diagnostic biopsies of DN calculated using equation (1) and presented here as a percentage. Six studies, including our own, provide data able to be analyzed to obtain the PPV of positive margins in diagnostic biopsies of DN. In each study, the PPV of positive diagnostic margins is less than 50%.

Residual nevus cells were described in 29 of 63 available excisional pathology reports. This illustrates a PPV of positive margins in diagnostic biopsies of DN of 46.0%. This value is similar to the findings of Maghari (44.9%) [2] and higher than those of Reddy et al. (33.0%) [3], Cohen et al. (31.2%) [5], Duncan et al. (29.4%) [6], and Strazzula et al. (18.2%) [4] (Figure 1).

**Figure 2:**
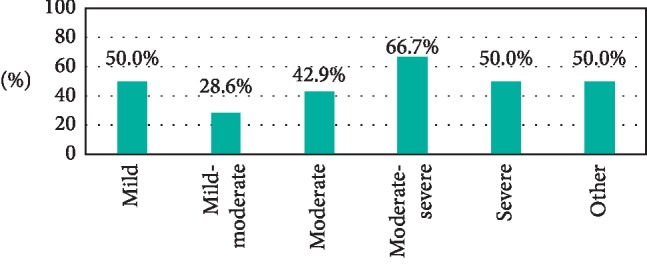
PPV and degree of severity. PPV here is stratified based on degree of severity of melanocytic atypia at diagnosis and presented as a percentage. In our patient population, positive margins were most predictive when a moderate-severe degree of atypia was diagnosed.

The PPV was also analyzed separately for each degree of severity of DN with residual nevus cells being found on excision in 50.0% of mildly DN (2 total excisions), 28.6% of mild-moderately DN (7 total excisions), 42.9% of moderately DN (28 total excisions), 66.7% of moderate-severely DN (6 total excisions), 50.0% of severely DN (6 total excisions), and 50.0% of other or unspecified atypia (14 total excisions) (Figure 2). The data's power is greatest for moderately dysplastic nevi as it was by far the largest sample size.

The underlying reason for the low PPV of positive margins in diagnostic biopsies of DN and the discordance between diagnostic biopsy and excisional findings should be researched further. It is possible that a postbiopsy inflammatory response may contribute to the dissolution or obscuration of any residual nevus cells. Another explanation may be that unaffected tissues may shrink disproportionately when compared with tissue in which nevus cells are present causing a diagnostic margin to appear positive. Blasdale et al. described tissue shrinkage of the histological margin of excisions of basal cell carcinoma and reported that tumor-free tissue underwent a disproportionately high rate of shrinkage when compared with tumor affected skin, making the affected cells appear closer to the margin than they really were [7].

Because the protocol for histopathological analysis of excisions of DN does not require tissue sectioning to exhaustion, it is possible that residual nevus cells in excisional specimens may be missed. We acknowledge that a limitation of our study was an inability to perform repeat sectioning on the excised tissue specimens for review and confirmation of the initial report. We also acknowledge the limited possible examination of the margin in diagnostic biopsies. Further research could be conducted with the inclusion of a second review of the tissues by dermatopathologists to determine if further sectioning does in fact change outcomes.

## 4. Conclusions

In October 2018, the Pigmented Lesion Subcommittee of the Melanoma Prevention Working Group published findings regarding the risk of melanoma in moderately DN excisionally biopsied but with positive histological margins and no subsequent re-excision [8]. A total of 467 moderately DN with positive histologic margins from 438 patients were evaluated and of these, no cases developed into cutaneous melanoma at the biopsy sites with a mean follow-up time of 6.9 years. They also reported that a rate of clinical recurrent pigmentation at the biopsy site of 1.2%. A consensus statement has been published stating that observation may be a reasonable option for incompletely excised moderately DN without clinical residual pigmentation [9].

In another large-scale retrospective cohort study Fleming et al. provided evidence in favor of clinical observation for biopsy-proven mild or moderately DN with positive histologic margins. Their data included 304 observed DN with a mean follow-up of 5.5 years. Of the 304 observed DN, only 10 recurred (3.3%), and only 1 patient (0.33%) developed melanoma following an excisional diagnostic biopsy [10].

Our study demonstrates the low PPV of positive margins in diagnostic biopsies of DN and is consistent with the Pigmented Lesion Subcommittee recommendation that DN of moderate severity or less may be clinically monitored. Our findings can be used along with their statement as evidence to help providers determine the best treatment plan for their patients. Specifically, our study lends further evidence to the conclusion that completely biopsied margin-positive DN of moderate severity can be safely monitored, as our data set's power was greatest was for the moderate severity group. In this group we observed a PPV of 42.9%, indicating that over half of postbiopsy excisions showed no signs of residual nevus cells. Even with positive margins on initial diagnosis, providers should exercise clinical judgement when considering management, especially when surgery may safely be avoided.

Adding our findings to those of other studies increases the body of knowledge surrounding DN and allows dermatologists to improve clinical management. We hope that quantifying the predictive value of positive margins in biopsies of DN will aid in the decision-making process when treatment options are considered, and this study can be a resource for providers. When the low risk of recurrence or transformation is considered along with the low PPV of a margin-positive diagnostic biopsy, we consider that many postbiopsy excisions of DN may be deferred if judged clinically appropriate, regardless of margin status.

## Data Availability

The retrospective clinical data used to support the findings of this study are restricted by the Midwestern University Institutional Review Board in order to protect patient privacy. The data used to support the findings of this study are available from the corresponding author upon request.

## References

[1] Tripp J. M., Kopf A. W., Marghoob A. A., Bart R. S. (2002). Management of dysplastic nevi: a survey of fellows of the American Academy of Dermatology. *Journal of the American Academy of Dermatology*.

[2] Maghari A. (2017). Dysplastic (or atypical) nevi showing moderate or severe atypia with clear margins on the shave removal specimens are most likely completely excised. *Journal of Cutaneous Medicine and Surgery*.

[3] Reddy K. K., Farber M. J., Bhawan J., Geronemus R. G., Rogers G. S. (2013). Atypical (dysplastic) nevi. *JAMA Dermatology*.

[4] Strazzula L., Vedak P., Hoang M. P., Sober A., Tsao H., Kroshinsky D. (2014). The utility of re-excising mildly and moderately dysplastic nevi: a retrospective analysis. *Journal of the American Academy of Dermatology*.

[5] Cohen L. M., Hodge S. J., Owen L. G., Callen J. P. (1992). Atypical melanocytic nevi. *Journal of the American Academy of Dermatology*.

[6] Duncan J. R., Purnell J. C., Stratton M. S., Pavlidakey P. G., Huang C., Phillips C. B. (2020). Negative predictive value of biopsy margins of dysplastic nevi: a single-institution retrospective review. *Journal of the American Academy of Dermatology*.

[7] Blasdale C., Charlton F. G., Weatherhead S. C., Ormond P., Lawrence C. M. (2010). Effect of tissue shrinkage on histological tumour-free margin after excision of basal cell carcinoma. *British Journal of Dermatology*.

[8] Kim C. C., Berry E. G., Marchetti M. A. (2018). Risk of subsequent cutaneous melanoma in moderately dysplastic nevi excisionally biopsied but with positive histologic margins. *JAMA Dermatology*.

[9] Kim C. C., Swetter S. M., Curiel-Lewandrowski C. (2015). Addressing the knowledge gap in clinical recommendations for management and complete excision of clinically atypical nevi/dysplastic nevi. *JAMA Dermatology*.

[10] Fleming N. H., Egbert B. M., Kim J., Swetter S. M. (2016). Reexamining the threshold for reexcision of histologically transected dysplastic nevi. *JAMA Dermatology*.

